# A narrative case study of an older disabled Muslim woman during the COVID-19 pandemic in the UK

**DOI:** 10.3389/fsoc.2024.1369188

**Published:** 2024-04-17

**Authors:** Amani Alnamnakani

**Affiliations:** School of Healthcare Sciences, Cardiff University, Cardiff, United Kingdom

**Keywords:** COVID-19, pandemic, ageing, disablism, racism, religionism, sexism, chronic illness

## Abstract

This paper explores the experiences and perceptions of Zora, an older Muslim woman living with a disability in the UK. Older disabled Muslim women in the UK often face multiple discriminations based on disability, age, gender, religious, and racial grounds and this has arguably been intensified by the COVID-19 pandemic. Drawing on multiple narrative interviews with Zora, this paper focuses on the intersections of disability, ageing, gender, race and religion within a particular social context during the COVID-19 pandemic in the UK. The paper describes the complex ways in which Zora experienced various modes of everyday disablism which were not related to the COVID-19 virus itself, rather the consequences of the movement restrictions associated with it. Much of the oppression and barriers she described were socially determined, both through direct discrimination, stares and prejudicial attitudes, and indirectly through stigmatization and an embodied fear of the reaction of others in public spaces. Nevertheless, Zora did not present herself as a victim. Instead she portrayed herself in affirmative terms, as a ‘brave’ woman who resisted and overcame daily social challenges and movement restrictions as part of working toward creating a more accessible, inclusive and age-friendly society. One that is inhabitable for herself and other older disabled women facing an uncertain future.

## Introduction

The evolution of COVID-19, declared a pandemic by the World Health Organisation (WHO) in March 2020, had a devastating impact on different people all over the world (United Nations, 2020; Yan et al., 2022). Following the second wave of the pandemic, which began on September 2020, several UK wide measures were implemented. For example, rules on face masks, social distancing, a staying at home order, cancelation of mass gatherings, school closures and larger lockdowns to control the spread of the disease, and mitigate the negative impacts of a crisis (BBC, 2020a,b). In this regard, disabled women, older women, and Muslim women have specific vulnerabilities as the pandemic produced new forms of vulnerability in addition to the vulnerabilities that existed previously.

Disabled women, often more intensely than women in general, have been cast in the collective cultural imagination as inferior, unfit, useless, undesirable, asexual and incapable of independence (Hanna and Rogovsky, 1991; Garland-Thomson, 2005). They face double discrimination on the basis of gender and disability disparities, these combine to produce poor outcomes such as lack of access to healthcare, low employment, education, low income levels, and fewer opportunities for vocational training (Schur, 2003; Ekblom and Thomsson, 2018; Schalk and Kim, 2020). Moreover, disabled women are vulnerable to sexual violence because of the stigma and prejudice associated with their disability. As such, they are among the most vulnerable and marginalized social groups of society (Dockerty et al., 2015; Ekblom and Thomsson, 2018; Kirk-Wade, 2023). Yet, Asian or Black disabled women face the triple oppression of sexism, racism and disablism and, as a consequence of these social categories, they are socially excluded and placed in an extremely marginalized position (Begum, 1992).

Older women are often expected to experience more disadvantages than older men (Yan et al., 2022). For example, it is suggested that a tension exists for older women in attempting to access employment, they often face a disproportionately increased risk of job loss due to automation and technological change as well as being subjected to gendered ageism (Sargeant, 2011; Yan et al., 2022). Yet, findings from a meta-analysis study revealed that older women were more likely to be abused than older men, and such gender disparity is more evident in non-Western populations than their Western counterparts (Ho et al., 2017). These aspects of discrimination, bias, and marginalization create barriers to older women’s social participation. Such barriers are further compounded for disabled older women who are systematically overlooked and underrepresented in initiatives, development policies, programmes, legislation and humanitarian efforts. However, ageism, ableism and gender inequality are further exacerbated by other forms of discrimination on the basis of religion, race, and migration status amongst other factors (UN Women, 2022).

For example, Jawad and Benn (2003) suggested that most Muslim women experienced double oppression, as a result of the culture of their community and the culture of their religion. In the community, Muslim women were amongst the targeted victims of anti-Muslim hate crime encountered by Muslims living in the UK because they wear one of the most recognizable symbols of Islam - the hijab (Jawad and Benn, 2003; Dodd, 2019). This symbol has become riddled with racial meaning such as ‘foreign’, ‘violent’, ‘oppressive’, and ‘misogynistic’ (Siraj, 2012; Selod and Embrick, 2013; Al Wazni, 2015). Taken together these stereotypes result in the belief that a Muslim woman’s body is incapable of Western ideals and values (Selod and Embrick, 2013), this explains why they also experience racial discrimination. Only 1.2% of Muslims in the UK described themselves as being of White British origin (Ali, 2015). Regarding the culture of their religion, Muslim women have traditionally been assigned for motherhood and homemaking roles. This is the case despite the fact that Islam states that such roles are neither exclusive nor inflexible (Islam House, 2013).

However, given that relatively few studies have examined the impact of the COVID-19 pandemic on older women, disabled women and also Muslim women in the UK, the literature used in this study was mainly from other countries. For instance, in a nationwide survey of older women in the United States, VoPham et al. (2022) found notable changes in lifestyle factors during the COVID-19 pandemic. Over half of older women reported less physical activity or exercise compared with before the pandemic, this may have both short and long-term effects on the health and wellbeing of these women (VoPham et al., 2022). In a large, population-based study of community-dwelling older women in Hong Kong, Yan et al. (2022) revealed that psychological abuse was the most prevalent form of abuse against older women during the COVID-19 pandemic, this was followed by financial or economic abuse and physical abuse.

According to the UN Women (2020), disabled women often face additional challenges in their attempts to flee violent situations, such as sexual exploitation and consequential economic deprivation, or to access protection orders or essential services which offer refuge, such as lockdown or quarantine. The COVID-19 pandemic resulted in an increased burden of domestic violence and unrecognized unpaid domestic labor for Muslim women (Safdar and Yasmin, 2020; Jailani, 2022). As a consequence they became the backbone of their family economy, acting as household managers, taking the main role in children’s care and education, and becoming a leader within the family, this caused anxiety and mental stress (Safdar and Yasmin, 2020; Jailani, 2022).

Although the pandemic illuminated the experiences of older women (VoPham et al., 2022; Yan et al., 2022), disabled women (UN Women, 2020) and Muslim women (Safdar and Yasmin, 2020; Cahyaningtyas et al., 2021; Jailani, 2022) in the world’s strictest lockdown, the intersections, convergences and the connections of these identities have been under-researched. Literature addressing the impact of COVID-19 in relation to gender, age, disability and religion identity, has largely overlooked the concerns of older disabled Muslim women and debates about these identities often take place in isolation from each other. Yet, these markers of difference need to be considered in relation to each other to bring to the fore the paradigm of intersectionality and to show that disability identity does not function in isolation from other social categories. Rather, they accentuate each other contextually and as a process.

Older disabled Muslim women, irrespective of impairment, are often disadvantaged on multiple fronts, they face discrimination in everyday life situations and within different contexts/societies (Hanna and Rogovsky, 1991; Turmusani, 2001; Jawad and Benn, 2003; Schur, 2003; Dossa, 2009; Sargeant, 2011; Alnamnakani, 2022). Discrimination against these women is not merely a function of the women’s subordinate position in society (Dossa, 2009), but is a result of the intersections of ageing, gender, race, religion and disability identity. While few studies have examined the experiences of Muslim women with disability across the world such as Hussain (2005) in the UK, Dossa (2009) in Canada, and Turmusani (2001) in the Middle East, there do not appear to be any studies which explore the everyday life and experiences of older disabled Muslim women during the COVID-19 pandemic in the UK. The goal of this study, therefore, is to address this gap in the literature. The study explores the lived experience of an older disabled Muslim woman, taking into account her racial background, in the context of the COVID-19 pandemic in the UK. In this study, Zora shares her story of what it is like to be subject to discrimination as a result of having multiple identities.

Based on multiple narrative interviews which took place over a period of 18 months, this study addresses how Zora makes sense of and copes with the challenges associated with her multiple identities during the COVID-19 pandemic in the UK. Yet, since there is neither a single method for narrative analysis nor the analysis of the intersectional identities (Nasheeda et al., 2019; Turan et al., 2019), it was necessary to develop the ‘narrative intersectionality’ approach, this aimed to craft an analytical tool that would be sensitive to the intersectional identities of the participant throughout her narrative.

Following a discussion of methodological approaches, the article explores Zora’s experience of her multiple identities in the context of the COVID-19 pandemic in the UK. This is covered in one subsection and is preceded by an overview of Zora’s upbringing and diagnosis: it discusses her experience of vulnerability whilst traveling by train, a public place where attitudes such as stigma, prejudice, and discrimination are derived from people’s beliefs and ideas related to disability, ageing, race, and religion. This is followed by a discussion of the findings and the conclusion of this paper.

## Methodology

For the purpose of this article, the present data includes a subset of information for one participant taken from an original study which explored the lived experiences of five disabled Muslim women of varying ages in the UK (Alnamnakani, 2022). The study, however, is not concerned with generalizing the result and is dedicated instead to developing an in-depth understanding. Including fewer participants enabled more in-depth information to be collected about the phenomena under exploration. In the context of this paper, the participant’s name has been replaced with a pseudonym.

This study utilises a narrative inquiry as a form of qualitative research, this focuses not only on *“individuals’ experiences*” but also on exploring *“the social, cultural, and institutional narratives within which individuals’ experiences are constituted, shaped, expressed, and enacted”* (Clandinin and Rosiek, 2007, p. 43). Narrative inquiry was selected for this study because of the role that it plays in giving a voice to silenced and marginalized people (Bruner, 1991; De Fina, 2015). It is a way of creating and sharing meaningful content from which people’s experiences can be transmitted (Muylaert et al., 2014). The term narrative carries many meanings and it is often used synonymously with story (Riessman, 2008), such as in this study.

Parker and Shotter (1990) described narrative as the verbal recounting of life events as a story. Narratives allow people not only to tell what happened in the past, but also to enhance people’s understanding of the present and possibly their future as well (Mattingly and Garro, 1994). Thus, people use narrative to understand and make sense of who they are and who they may become, by reference to where they have been and what they have been throughout their life. Selecting narrative inquiry in this study enabled me to focus on personal narrative, to start with participant’s situation from the onset of her disability up until the time when she tells her story. This in turn enabled me to view the whole web of this woman’s experiential reality of living with her multiple identities, particularly in a crisis time such as the COVID-19 pandemic.

A purposeful sampling was used to recruit participants with different experiences, ages, backgrounds and educational levels in order to reflect the diversity of these disabled Muslim women (Patton, 1990). The eligibility criteria included Muslim women who were residing in the UK, were at least 18 years of age, spoke English or Arabic, and self-identified as disabled. The rationale for selecting the narrative of one participant in this article was that her experiences demonstrated how approaches to older age and disability intersect with gender, race, and religion, and how these have been intensified by the COVID-19 pandemic. Once ethical approval had been granted by Cardiff University, School of Healthcare Sciences Research Ethics Committee, participants were recruited from various sources including disabled people’s organizations, feminist organizations, Islamic mosques, Muslim women’s networks, women’s groups, as well as personal Instagram accounts and snowball sampling (Alnamnakani, 2022).

Data were collected through multiple narrative interviews combined with semi-structured interviews, observing nonverbal communication and note-taking data. Narrative interviews are considered as a form of in-depth interview which emerges from the life stories of the participants and their context (Jovchelovitch and Bauer, 2000). According to Muylaert et al. (2014, p. 184) “*they allow the deepening of research, the combination of life stories with socio-historical contexts, making the understanding of the senses that produce changes motivates and justify the actions of possible informants*.” Hence, using narrative interviews can lead to a better understanding of the participants’ perceptions and their social world.

According to Anderson and Kirkpatrick (2016) narrative interviews can be conducted together with semi-structured interviews and observation. Semi-structured interviews seek to uncover the person’s lived experience (Walker, 2011). It permits the researcher to use open-ended questions which then allows the participants to respond in their own words and to describe the richness of their personal experiences in relation to their context (Bryman, 2012; Polit and Beck, 2014). Three in-depth interviews were conducted with the participant in this study. Each interview lasted between 45 and 90 min, depending on the interest, willingness and availability of the participant. The initial interview was conducted in-person, but the following interviews were conducted via virtual platforms (Zoom video meeting) due to COVID-19 restrictions.

Observing nonverbal communication and the effect of the context was used during the interviews to enrich the data with the meanings that are taken for granted. For example, facial expressions, gaze, silence, gesture, posture, tone of voice and other nonverbal interactional communication and body languages that could not be captured in an audio recorded interview. Interactions which occurred during the interview were observed and then reported in detailed notes immediately after each interview. The note-taking included nonverbal communications, information given by the participant in the concluding talk when the recorder had been switched off, what was seen/observed during the interview and any impact that the presence of the researcher may have had on the situation.

The data analysis methods were comprised of manually transcribing the recorded interviews and developing the ‘narrative intersectionality’ approach to analysing and interpreting the collected data. The ‘narrative intersectionality’ approach, was developed based on Chadwick (2017), and Blackie et al.’s (2019) concept of narrative intersectionality. The aim was to craft an analytical tool that would be sensitive to the intersectional identities of the participants throughout their narratives. The ‘narrative intersectionality approach’ conceptualises a participant’s narrative as multidimensional and comprised of multiple analytical layers concerning multiple identities. The story is co-constructed between the participant and other actors within a particular social context and at specific intervals in her life.

The analytical process using the ‘narrative intersectionality’ approach included 7 steps, these were organized into 4 stages. The first stage involved 3 steps. Step 1 was the initial reading of the transcript and broad coding, and step 2 was re-reading and making initial notes on a story. Step 3 was listening to the participant’s voice in her own words and reconstructing a story, the codes from step 1, as informed by notes from step 2, led to the development of emerging plots or storylines, outlining the main elements of the participant‘s story. These first 3 steps were descriptive, focusing on understanding and developing the participants story and seeking to develop its initial plots.

The second stage was interpretive, aiming to produce meaning by engaging the emerging storyline with the related theory. It discussed the data with theory, and the theory was then used to fill the meaning-gaps in the emerging storylines and explain the story. The third stage involved 2 steps, further data collection and repeat process. These steps were interactive and aimed to interweave between the data collection and analysis process. This process was reiterated until a meaningful narrative was constructed. The fourth stage involved writing-up, aiming to refer to the conceptual framework which relied on the intersectionality theory to illuminate the complexity of the participant’s experience and engage the co-constructed narrative in a critical discussion with the relevant theories.

According to Evans (2016) and Samuels and Ross-Sheriff (2008), intersectional studies should recognize the ways in which social context can affect social inequalities, particularly the way in which historic, economic and political conditions can shape participants’ experiences, and can cause resistance to marginalization and exploitation across various contexts. In other words, intersectionality aims to address the manner in which sexism, ageism, racism, class and other systems of discrimination create inequality. As such, intersectionality calls for women to be viewed as whole beings; to recognize that not all women experience their womanhood in the same way. Many women face multiple forms of oppression but not all are rendered powerless (Samuels and Ross-Sheriff, 2008). It was felt important, however, to push this concept further and suggest that individually disabled Muslim women in the UK experience their multiple identities and various interlocking oppressions differently in different social contexts at different intervals of their lives, such as during the COVID-19 pandemic.

To enhance the credibility of this study prolonged engagement with the participant was used to support rigorous data collection, and gives a better understanding of the research context from the participants’ point of view (Creswell and Miller, 2000; Shenton, 2004; Ryan et al., 2007). Data collection occurred through meeting and interviewing the participant on three occasions over a period of several months. There was at least a 4–8 month period between each interview and it was essential to maintain contact with the participant during this time to reduce attrition. Maintaining engagement with the participant facilitates a building of trust in the relationship. Mmeber checking also occurred during each interview. Shenton (2004, p. 68) termed the provision of the accuracy of the data during the interview course as “*on the spot*” check. I re-framed questions or expanded on them using probing questions to explore issues the participants were describing in more detail. I also feedback to the participant to ensure that the constructed narrative reflected her perspective.

With regard to the ethical considerations, the participant was offered time to ask questions and consider her decision to take part in the study before signing the consent form. The participant voluntarily consented to sign the form which involved the following point: (I understand that the direct quotations from my story and the findings and potentially secondary analysis of the findings and associated data from this study may be presented at conference and published in scientific journals. I understand that these will be used anonymously and that no individual respondent will be identified in such report).

With respect of issues of anonymity, pseudonym was allocated and some of the minor details about the participant were changed. Some personal and demographic information was concealed such as using an approximate rather than exact age of the participants. In addition, anonymity was further maintained by omitting some of the data in the interview transcript or by judging what should be left out in the analysis based on the interview.

## Findings

The following findings aim to highlight how an older Muslim woman experienced life with disability, how she perceived herself in her local context and how she coped with the challenges she faced during the COVID-19 pandemic in the UK. This, however, does not mean that the narrative provides an exhaustive account of the participant’s experiences of living with disability. More specifically it foregrounds some parts of this woman’s experiences with disability in her local context and in a specific time frame of the pandemic, where other parts of her experience are not given the same attention.

Meanwhile, the narrative also brings to the fore the paradigm of intersectionality to show how differences of gender, age, race, religion, and disability accentuate each other. This should be understood contextually rather than viewing disability as a master category under which other categories are subordinated. Consequently, the narrative that follows does not make any claims for the generalisability of older Muslim women’s experiences of living with disability, rather it highlights the experience of a specific older disabled Muslim woman in the context of the COVID-19 pandemic in the UK. Yet, for the production of generalization, there are a number of issues that could be explored with social groups of disabled women. Several stories of other disabled women in the UK remain unknown, untold and unheard.

The findings tell Zora’s story, an older disabled Muslim woman in her 60s. Zora is a retired nurse who is married and had two children from a previous marriage. In 2003, Zora contracted HIV from her first husband. Following the HIV diagnosis Zora was diagnosed with a number of mental health conditions, these included bipolar and borderline personality disorder. At the same time, Zora described practising her religious traditions more, and began to wear a niqab (a veil covering all of the face apart from the eyes). In 2009, Zora developed chronic back pain, osteoarthritis, chronic IBS (irritable bowel syndrome) and stomach ulcers. She was also diagnosed with fibromyalgia after suffering long-lasting pain, which affected her mobility. As a consequence, Zora had to use crutches to walk short distances both indoors and outdoors.

Zora lived with her mother as her husband was working in another city. Throughout her narrative, Zora described how she was frequently stared at because of the way she walked and because of other categories of her identity, such as being “*Black”* and a “*lady with a niqab.”* In spite of this, she described how she tried to cope with people staring, as an older disabled Muslim woman with a niqab, in an attempt to carry on her daily activities independently and feel a sense of belonging, but this was not always successful. Zora described feeling “*vulnerable”* to sexual harassment on the train due to general hostility toward body differences, one of her everyday life contexts during the COVID-19 pandemic. The discussion section describes how the narrative provided a response to the objectives of this study and illustrates how the participant in this study resisted and overcame daily social challenges to recreate a better world for herself, other older disabled Muslim women, and the next generation of disabled people.

### Zora’s upbringing and diagnosis

When Zora was 6 years old, and whilst playing with her siblings, she recounted that they used to have “*a very short person”* walking around in their neighborhood. As children, Zora described how they tended to stare at this person. In response to their behavior, Zora stated that her

“mum used to say, don’t stare, don’t laugh at people because you don’t know what’s your future is going to be, so we were raised like that”.

Staring is fastening one’s eyes on someone as a way of strongly expressing response and reacting to others (Garland-Thomson, 2006, 2009). It is a more sustained form of looking than glimpsing, gazing and other forms of normative looking, which registers intense interest and endows it with meaning. That interest can take many forms ranging from curiosity, wonder, disgust to hostility (Garland-Thomson, 2006). Zora described how her mother taught her and her siblings at an early age that anyone had the potential to become disabled or look different, including them, in the future. This familial context encouraged Zora to volunteer her time after school and at weekends to look after older people, *“the disabled, people with learning disabilities, children, [and] teenagers with learning disabilities.”* Thus, she became a volunteer “*carer”* at the age of 16 years. Her experience of looking after people developed further during her university placement, prior to working as a nurse in the NHS. She said:

“I worked for the NHS for 30 years. I’m proud of the things that I’ve contributed in the past”.

Zora was proud to work at NHS hospitals where she looked after people for many years. She told me that she wanted to do a postgraduate course to specialize further in mental health nursing, but she felt that the mental health problems due to her first marriage prevented her from doing so. Zora found it difficult to talk about her own mental health.

Zora: “I wanted to specialise, and I wanted to do mental health, but I have a mental health issue”.

Amani: What mental health issue?

Zora: “So, basically a bipolar and then I have a borderline personality disorder, but I think, I have, I was married, I’m married now, but I was married before to someone else, and he was an HIV positive”.

In 1997, Zora got married. She stated that her first husband was HIV positive, but he did not inform her about his health condition when they got married. “*He kept it secret,”* she said. When Zora was expecting her second baby in 2003, she “*started to feel really sick”* and visited the hospital. She explained that she had a medical check, including a blood test, but they told her:

“You are okay. And then, two weeks later they called: *‘you have to come back to the hospital now, now, now, now’* [emphasis by Zora], so they can stop the baby from getting this [HIV] condition, so basically then I was diagnosed with post-traumatic stress disorder because of what he did”.

When Zora was first diagnosed with HIV, she described being “*very stressed out”*. The challenge was not merely accepting HIV and living with it but also what to do with her husband. She told me that her ex-husband was arrested and was taken to court. Going to court for the trial was stressful for Zora, and the struggle was evident in her words when she spoke:

“The trial was like, made me, I was, I was very stressed out, they said, you have post-traumatic stress disorder, but I forgive him, and I leave it to Allah [God]”.

Following her diagnosis with post-traumatic stress disorder, Zora described how she resorted to two strategies: forgiveness and reliance on Allah. Worthington and Scherer (2004) stated that forgiveness could be used as an emotion-focused coping strategy to reduce a stressful reaction to a transgression. Thus forgiveness was a form of coping used by Zora to help alleviate stress caused by the traumatic events described above, and which affected her mental health. Secondly, Zora also credited her faith in God for getting her through that stressful time. She described laying her trust in “*Allah”* and leaving the rest to him because she believed nothing is difficult for Allah. Accordingly, after obtaining a divorce, Zora explained how she tried to start a new life.

“I moved and stayed with my two children. I just started to practice my deen [religion] more. I started wearing a niqab … so now I wear a niqab, but I’m still the same person who goes to the shop, says good morning, and then the person there goes, ‘good morning, how are you?’, ‘I’m fine;’”

Underlying her action of continuing to work, going to the shops and moving with her children was a vision of life where being a HIV positive Muslim woman would not mean living in isolation. These actions enabled Zora to subvert the stigmatized identity of a Muslim woman who had HIV both for herself and others. Zora also described remaining *“the same person”* who loved interacting with others in the community. She believed that HIV and the niqab did not change who she was in terms of her social behavior and interaction with other people in the community. In addition to HIV, Zora also developed chronic back pain, osteoarthritis, chronic IBS and stomach ulcers between 2009 and 2011. She described being in a lot of pain due to these medical conditions:

“The pain is like I’ve been stabbed with a knife and I would stay on this couch, and I can’t move, I can’t get up for salah [prayer], I can’t get up to go to the toilet, and I can’t even umm eat because of the pain”.

The pain did not only affect Zora’s everyday life activities, such as eating, toileting and praying; it also affected her ability to work. Zora had worked until 2012 when she retired on health grounds. “*That’s how bad it was all the pain in my body,”* she said. Therefore, Zora believed she was disabled from the time she began to experience the pain in 2009, this started several years after the HIV diagnosis. She explained that her support worker tried to help her apply for a disability allowance, but she did not qualify at that time.

“They said that just because you are in pain doesn’t mean you can qualify for it, and PIP, which is personal independent payment, concentrates on your mobility”.

Zora did not qualify for the PIP, to help her with the extra cost of living with a disability, until fibromyalgia affected her mobility, a diagnosis which was confirmed in 2015. She described how pain due to fibromyalgia, combined with osteoarthritis, chronic back pain and stomach ulcers, resulted in her having to use crutches and “*start to be very slow and then every step agony”*. As such, the following section describes Zora’s experience of her identity as an older Black disabled woman who wore a niqab and used crutches in one of her everyday life contexts during the COVID-19 pandemic: public transport.

### On the train; “I realised I’m vulnerable”

During our conversation, Zora spoke of her feelings about the way she walked and how people reacted toward her within her local community. She said:

“I feel awkward because of the way I’m walking, it’s like I’m walking down a hill, and the way I’m walking with my crutches is extremely slow, and people are staring at me, and I don’t know why they’re staring at me, I don’t know if they’re staring at me because of that or because I’m Black, or because I’m Muslim, or because of my disability, or because my age [laugh]”.

At the heart of Zora’s words is the matter of her body appearance. Walking slowly, older adult, Black skin, niqab, and crutches – all of these are body characteristics and embodied practices. She began by describing her walking as *“extremely slow,”* she believed this to be a primary reason for people staring at her. Garland-Thomson (2017) argued that stare indicates a disabled identity about the body when markers, such as Zora’s crutches, are conspicuous to people. In this case, Zora’s body disrupted the body norm expectations and invited the stares that constituted her as other. But Zora also said she was unsure if her disability was the only reason for being stared at. Her uncertainty was complicated by the presence of other categories of her identities, which she believed invited staring; namely, being an older, Black and Muslim woman with a niqab.

An older women could be stared at, as Cecil et al. (2022) suggested, because they are subject to both sexism and ageism, and consequently be stigmatised as their appearance looked dissimilar to societally favored youthfulness. Wilkinson (1969, p. 191) referred to situations in which Black people find themselves receiving the “*Black hate stare”* in various forms in public, such as frowns and glances. Zempi (2020) reported that Muslim women in the UK who wore the niqab experienced stares from strangers in public places due to their visible religious identity and gender performance. In the following extract, Zora explained how she responded to a staring incident on the train during the COVID-19 pandemic:

“When I was on the train, I was sitting in a carriage where there was no one else and four rows ahead of me there was a man, this man came behind me by my seat and then he just didn’t talk but WOOH [loud voice expression], like that. I just went Ahh [panic expression], he was holding his private part and staring at me. When I saw him holding his private part, I turned to look through the window, and when I was looking through the window, I couldn’t see if I can see anything, I stared at the window, and for me, it took like an hour, but it would be three minutes or so, and then while I was staring he was laughing. After three minutes of staring at the window, I finally could see his reflection. I don’t know whether because we entered to the tunnel and there was dark, and you can see, and it’s like a mirror now, it’s not like you’re looking to trees or anything, and he was shaking his private part and staring up and down at me”.

Blocking a person’s path, making sexual gestures through touching oneself in the presence of another person, and looking at a person up and down, are all non-verbal examples indicating Zora’s experience of sexual harassment on the train. Sexual harassment involves unsolicited and rejected verbal or nonverbal sexual gestures that happen in different ways by a male stranger toward a female, solely based on her sex, in a public place (Laniya, 2005; Ludici et al., 2017; Mason-Bish and Zempi, 2019). However, to understand any woman’s experience, Davis (1994) and Mason-Bish and Zempi (2019) emphasized the importance of thinking of her as embodied and not as a person experiencing sexual harassment on various, nonintersecting axes, and then thinking about the particular meanings assigned to that embodiment in her context.

Dalton et al. (2021) and Martin et al. (2006) found that non-White women were more likely to have experienced sexual assault, to have been judged unfairly and blamed for the occurrence of the assault. Disabled women were among those who suffered most from sexual crime in the transport environment as their impairment limits their ability to defend themselves. This may lead the perpetrator to feel that disabled women would be relatively powerless to resist (Martin et al., 2006; Casteel et al., 2008; Ludici et al., 2017). Older women, however, face particular barriers to disclosure of sexual assault, this results in their experiences remaining hidden (Fileborn, 2017).

On the other hand, Haddad et al. (2006) argued that Muslim women had been historically portrayed as either hypersexual women or silent images of oppressed victims of male brutality. Yet, women who wear the hijab, in particular, were subjected to sexual harassment based on stereotypes about their gender, religion and race. This positions their bodies as passive and incapable of resisting male offence (Alimahomed-Wilson, 2017). In this regard, wearing the niqab marked Muslim women out as more readily visible and as soft, easy, and convenient targets for attack (Mason-Bish and Zempi, 2019). Zora acknowledged that she was vulnerable, as an older Black disabled Muslim woman with a niqab who was traveling alone in an empty train. Measures to restrict movement had been introduced by authorities during the pandemic, this had led to a drop in passenger demand (Marra et al., 2022). As a result she described her thoughts and her response to the man’s behavior whilst she was traveling on the train:

“I was just holding one hand on my bag and the other hand on my crutch. With my crutch, I thought in my head should I hit him with my stick? What if I hit him with a stick, he might get hold of the stick and beat me with the stick, so I can’t hit him, I was frozen, and then he just walked away. So then I had my suitcase, the next stop I got off, and it took like maybe 20 minutes to get to the exit, for somebody that would take them five minutes. And as soon as I saw the person behind the window at the station, I burst into tears and I said please I need to report something to the police, and then I thought I should never get on the train again on my own for the rest of my life. Although I video what happened to prove this, and I thought maybe the video would be evidence to the police, and that’s when I realised I’m vulnerable, it hits me like a COVID”.

Zora’s expression “*I was frozen,”* meant she resorted to the fight, flight and freeze response in the presence of a stressful threat (Samra, 2019). At first, Zora described thinking about the fight response. She thought of hitting the man with her crutch, but realised she could not protect herself if he took the crutch and started beating her. Bearing in mind that he stood by her seat and she could only walk slowly on her crutches, due to her age and physical impairment, neither fight nor flight and escaping from him seemed possible. Thus, Zora described finding herself in a situation where she could not act or move at all; she froze until the man walked away.

According to Owen (2021), reports of sexual harassment on public transport in the UK increased by 63% in 2021 when compared with the figures for 2019, prior to the pandemic. Yet some women were still hesitant to report crimes, this was due to their mistrust of the police and the way age shapes the experiences of older victims of sexual harassment (Lazar, 2020; Owen, 2021). On the contrary, Zora did not present herself as a passive victim in the face of the perpetrator. Zora recorded a video during the stressful few minutes of her experience of sexual harassment so she could use this as evidence in police investigations, the carriage was empty and there were no witnesses. Then, when she managed to reach assistance at the station, she immediately reported the incident.

Concerning COVID-19, a report by OECD (2021) demonstrated the fact that the virus had a devastating impact on people’s physical health. It also showed that the crisis affected people who were already struggling the hardest, such as the ethnic minority community. In the same way Zora described how being alone and vulnerable, due to the pandemic movement restrictions, had strongly affected her well-being. On reflection, however, Zora referred to herself as a strong and brave person. She said:

“I don’t feel vulnerable in general before that day and thought I was a strong person who is brave, I’m not afraid of anyone, and then I realised that because I froze that means that I’m not. Ah, but I videoed it”.

For Zora, being a strong person did not mean having physical strength. Rather, it meant having the confidence to make choices, have freedom of mobility, and travel independently without requiring assistance. This was despite having a physical impairment which limited her mobility and resulted in her needing crutches. However, this incident placed Zora in a socially vulnerable position, encapsulated in the case of sexual harassment that lowered her self-esteem and made her feel weak and devalued. Swinton (2012) argued that belonging goes to a deeper level than inclusion. The term ‘inclusion’ means that all people should feel welcomed and incorporated into the fabric of their society without any kind of restrictions or limitations (D’Eloia and Price, 2016). Thus, to be included, Zora just needed to be present so that the transport system fulfilled some social or legal standards of inclusion and diversity. On the other hand, belonging can follow from broader environmental cues that indicate one’s identity is compatible with, or appropriate in, a given context (Slepian and Jacoby-Senghor, 2021). As such, to belong Zora needed to be seen as a valued human being and feeling able to be her true self.

According to Wilson and Van Antwerp (2021), having a psychological sense of belonging within a particular social context is essential for positive psychological and performance outcomes. They found that women in an under-represented group were particularly vulnerable to not belonging as their minority status, based on their gender, race, social class, religion or other under-represented status, can contribute to a negative feedback cycle associated with unmet belonging needs (Wilson and Van Antwerp, 2021). Zora described being a “*vulnerable”* commuter within the transport system during the COVID-19 pandemic, her belonging involved not only making the transport environment physically accessible, but also re-emphasising the need for a monitoring system which would make public transport safe for her as an older, disabled and Muslim woman.

As such, Zora believed she was vulnerable, as an older disabled woman she could not control these situations and could not shield herself adequately from their consequences. Older women are in general easily victimized and cannot defend themselves due to their reduced mobility and physical impairment (Block, 1983). In addition, Arstein-Kerslake (2019) and Scully (2014) affirmed that disabled women are at greater risk of experiencing specific types of harm or being a victim of hate crime or abuse.

Scully (2014) identified two types of vulnerability in relation to disability: inherent, and contingent vulnerabilities. Inherent vulnerabilities are those which are often due to physiological or biological characteristics directly resulting from the impairment itself. For example, Zora’s impairments are associated with mobility problems such as poor balance and risk of fall when walking. Contingent vulnerabilities are those which are directly created by the social environment, they are contingent upon social and cultural responses to embodied differences which instigate and perpetuate situations and create vulnerability (Scully, 2014).

For example, an environment, such as public transport, that has not been made emotionally safe (the visceral feeling of being accepted for who you are and what you feel and need) to use for Zora, who used crutches, needed to walk slowly and required another person to guard her during attack. This environment left Zora vulnerable to the perpetrator, because when she was faced with the need to feel secure or escape safely she was dependent on the presence of another person or other passengers on the train. However, the train was empty as many people worked from home and avoided travel during the coronavirus pandemic. Thus, the perpetrator held power in this situation, and she believed that he might abuse that power. The perpetrator’s power was demonstrated through using some tactics to control Zora by intimidating, threatening, and causing fear by blocking her way. The utilization of these tactics, according to Crowley (2022), could place a heavy burden on the female victim based on the particular features of the setting and the characteristics of the victim. This is the vulnerability that is contingent on an inaccessible environment (Arstein-Kerslake, 2019).

If the public transport were made accessible with automated surveillance, older/disabled passenger alarms and other appropriate mechanisms to detect parameters such as unsafe behavior, then Zora might be able to use the train independently and would not need to depend on another person to guard her. To put it another way, the railways provide emergency buttons in the toilets of trains. Similarly, safety alarms could be provided on the train’s carriage for disabled and vulnerable people to draw the train crew’s attention to emergencies. Zora would, therefore, not be vulnerable to the potential for other people to use their position of power and to harm her. But against a background of such vulnerability, Zora was inherently and contingently vulnerable in the context of public transport.

Whilst it might not be possible for changes in the social environment to fully negate the impairment-related inherent vulnerabilities from the lives of disabled people, contingent vulnerabilities could, in principle at least, always be altered (Scully, 2014; Arstein-Kerslake, 2019). Thus, to eliminate her vulnerability, Zora described her decision not to *“get on the train again on [her] own for the rest of [her] life”*; this decision restricted Zora’s autonomy by limiting her independence in traveling alone. “*Under such circumstances, any inner reflections on who one is are eclipsed by the external definition of what one is in the eyes of others”* (Jackson, 2002, p. 68). Zora wanted to live in a way that positively acknowledged the difference of her age, religious tradition, physical ability and gender-based identity, but this was not possible in every situation.

Zora’s experience on the train reflected the ecological approach to disability. According to Nathan and Brown (2018), the ecological approach provides a useful framework for modeling dis-ability. They argued that disability cannot be reduced to the experience of an individual, caused by biology, society, or a combination of both because disability is not a property of an individual. Disability is only part of the story which cannot be analyzed independently without including the environmental features of the underlying context (Nathan and Brown, 2018). That is, the disability itself is not a disease or impairment existing within Zora’s body but only exists in the gap between her capabilities and the demands of the environment and context. In other words, Zora had the physical potential to travel independently, but when the perpetrator threatened her there were no measures on the train to ensure her safety, thus the person-environment interaction created a disability.

## Discussion

Much of the attention so far has focused on the implications of the COVID-19 pandemic on a single group of women, such as older women (VoPham et al., 2022; Yan et al., 2022), disabled women (UN Women, 2020) or Muslim women (Safdar and Yasmin, 2020; Cahyaningtyas et al., 2021; Jailani, 2022). However, the experiences of older disabled Muslim women has not been given the same attention. In this study, older adult, gender, race, religion and disability augmented one another in the participant’s display of herself to form a spectacle of embodied otherness that is simultaneously sensational and pathological. Zora, therefore, shared her story to demonstrate how she coped as an older disabled Muslim woman with a niqab, one who is independent and “*brave”* despite the challenging times presented by the pandemic restrictions.

Zora’s narrative touches upon her feelings of exclusion and vulnerability in one of her everyday contexts during the COVID-19 pandemic. Zora located her narrative in the context of public transport to highlight how one can be an actor and also a person who is acted upon. In this sense, she suggested that the bridge to accommodate her differences could be crossed through a context-specific sense of humanity implying respect for diversity as an outcome variable to crisis management, and ability to promote genuine harmony throughout the wider society.

Zora did not want to change her body or herself; she wanted to change the collective negative perceptions of old, disabled, ethnic and Muslim women as members of minority groups, particularly, during the COVID-19 pandemic (Armstrong and Watson, 2021; Walubita et al., 2021; Claus et al., 2023). Zora wanted people to recognize these facets of perception as socially constructed barriers and not as inevitable or natural. Accordingly, she did not only resist defining herself in terms of the stereotypes and preconceptions, observable in wider society, but insisted that another socially positive identity was appropriately descriptive of her. For example, she commented on how “*brave”* she was when she videoed the incident and reported it, she did so in order to support communities in developing solutions that would enhance vulnerable women’s safety, particularly during crisis times.

By portraying her life in this way, Zora asserted her agency in the sense that her resistance operates across the individual and collective levels and is enacted through self-reflection coupled with action (Gabel and Peters, 2004). Through these acts of resistance she celebrated a unique version of herself, as an older disabled Muslim woman, which she reconstructed from the perception of society about her identities as invisible, negative, passive, oppressive or without agency as described by Amini and McCormack (2021), and Turmusani (2001). Zora described being discriminated against and feeling vulnerable due to stigmatization which excluded her and set her aside as less important due to her multiple identities, it is an example of a situation which Oni-Eseleh (2021) described as social rejection.

The elements of social rejection which Zora experienced included being stared at, stereotyping based on cultural beliefs and denial of access to equal opportunities within her community. Knowles et al. (2014) found in their study that social rejection motivated people to distance themselves from the sources of rejection. In the same way, Zora reported that she would not use the train again on her own. This was due to her fear of people’s unpredictable reactions to her multiple identities. This may lead to lack of functioning and increased dependency, with a potential negative impact on her health and wellbeing. Adopting this coping mechanism, however, brings to the fore the idea that the goals of the stigmatisers are achieved and the unjust world is left intact.

As such, a social life was no longer hers to participate in and enjoy independently, it was dependent on how other people would perceive and react to her, also the availability of someone to accompany her. To put it in another way, Zora expected to arrive safely at her destination. Instead, she experienced a stressful situation and performed painful physical activities (i.e., carrying her suitcase while walking slowly with crutches to get to the nearest exit where she could get help) in her efforts to avoid such hostility, in the same sense as discussed by Block (2020) regarding the precarity of spirit when a person’s body and mind are under extreme stress due to a hostile living environment.

However, the aim of using the narrative intersectionality approach for this case study was to bridge the boundary between the medical model of disability and the social model of disability. Research has shown that healthcare students initially adhere to the medical model of disability, this views disability as something that must be fixed or cured in order for the disabled person to better fit into society and live a full life (Hirschmann, 2012; Cuff et al., 2016). I would argue that focusing on the body, as a disabled body needing a cure, can undermine the social issues that disabled people experience.

On the other hand, the social model of disability puts the onus of change on society, saying that inability to participate fully in social interaction is intrinsic to the society that is structurally, attitudinally and systemically inaccessible (Cuff et al., 2016). Yet, focusing exclusively on the social barriers in society obliterates the body from view, it overlooks the pain and suffering caused by the physical impairment that cannot be addressed through accessible society (Hirschmann, 2012).

As Shakespeare and Watson (2001) argued, the distinction between impairment and disability can be demonstrated by understanding where impairment ends and disability starts. While impairment is often a cause of disability, disability may itself produce or exacerbate impairment (Shakespeare and Watson, 2001). Therefore, incorporating the narrative intersectionality approach can develop a mutually beneficial integration of the two models, it creates a critical framework for discussing the medical and social factors that shape the individual experience of disability represented in the participant’s account. It can also transform practice for social change and view disabled people as whole beings: to recognize that not all disabled people experience the same multiple identities in the same ways; many encounter multiple forms of oppression, but not all are rendered powerless (Samuels and Ross-Sheriff, 2008).

## Conclusion

This paper provides an insight of the intersected experience of aging, disability, gender, religion and race for a specific older disabled Black Muslim woman during the COIVD-19 pandemic in the UK. The paper explores the strategies that the participant used to combat the deleterious effects of her multiple identities and strengthen her sense of agency in a lockdown world. Engaging with the narrative of this woman, this paper argues that the participant in this study endured a challenging context where social barriers related to her multiple identities left her not only unable to integrate but, more importantly, feeling vulnerable within the context of her everyday life. Her vulnerability was not related to the COVID-19 virus itself, restrictions imposed during the pandemic made her vulnerable in terms of her personal safety and wellbeing, not only in terms of health outcomes. To sum up, the present findings are of significance for understanding the long-term consequences of the COVID-19 pandemic restrictions on people living with multiple identities, such as older disabled Muslim women. To optimally serve them, attention should be given to their lived experience, daily social challenges and the lasting impact of the COVID-19 restrictions on their wellbeing, social lives, lack of freedom and independence, particularly if future lockdowns were to occur.

## Data availability statement

The datasets presented in this study can be found in online repositories. The names of the repository/repositories and accession number(s) can be found in the article/supplementary material.

## Ethics statement

The studies involving humans were approved by the Cardiff University, School of Healthcare Sciences Research Ethics Committee. The studies were conducted in accordance with the local legislation and institutional requirements. Written informed consent for participation in this study was provided by the participants’ legal guardians/next of kin. Written informed consent was obtained from the individual(s) for the publication of any potentially identifiable images or data included in this article.

## Author contributions

AA: Writing – original draft, Writing – review & editing.
